# Empirically based comparisons of the reliability and validity of common quantification approaches for eyeblink startle potentiation in humans

**DOI:** 10.1111/psyp.12545

**Published:** 2015-09-15

**Authors:** Daniel E. Bradford, Mark J. Starr, Alexander J. Shackman, John J. Curtin

**Affiliations:** ^1^ Department of PsychologyUniversity of Wisconsin–MadisonMadisonWisconsinUSA; ^2^ Department of Psychology, Neuroscience and Cognitive Science Program, and Maryland Neuroimaging CenterUniversity of Maryland, College ParkMarylandUSA

**Keywords:** Startle blink, Analysis/statistical methods, EMG, Anxiety

## Abstract

Startle potentiation is a well‐validated translational measure of negative affect. Startle potentiation is widely used in clinical and affective science, and there are multiple approaches for its quantification. The three most commonly used approaches quantify startle potentiation as the increase in startle response from a neutral to threat condition based on (1) raw potentiation, (2) standardized potentiation, or (3) percent‐change potentiation. These three quantification approaches may yield qualitatively different conclusions about effects of independent variables (IVs) on affect when within‐ or between‐group differences exist for startle response in the neutral condition. Accordingly, we directly compared these quantification approaches in a shock‐threat task using four IVs known to influence startle response in the no‐threat condition: probe intensity, time (i.e., habituation), alcohol administration, and individual differences in general startle reactivity measured at baseline. We confirmed the expected effects of time, alcohol, and general startle reactivity on affect using self‐reported fear/anxiety as a criterion. The percent‐change approach displayed apparent artifact across all four IVs, which raises substantial concerns about its validity. Both raw and standardized potentiation approaches were stable across probe intensity and time, which supports their validity. However, only raw potentiation displayed effects that were consistent with a priori specifications and/or the self‐report criterion for the effects of alcohol and general startle reactivity. Supplemental analyses of reliability and validity for each approach provided additional evidence in support of raw potentiation.

Potentiation of the defensive startle reflex in the presence of a threatening stimulus relative to a neutral, nonthreatening stimulus is a well‐validated translational measure of negative affect used in affective and clinical science (see Grillon & Baas, 2003; Vaidyanathan, Patrick, & Cuthbert, 2009, for reviews). Widely accepted guidelines exist for recording and measurement of the startle response in humans (Blumenthal et al., 2005; Stern, Ray, & Quigley, 2001). Recommendations also exist for the quantification of startle modification broadly (e.g., Berg & Balaban, 1999) and the quantification of prepulse inhibition specifically (e.g., Blumenthal, Elden, & Flaten, 2004; Hawk & Cook, 2000). However, empirically based guidelines for quantifying startle potentiation in humans have yet to be established despite the frequent use of this measure in clinical and affective science. This omission is nontrivial because commonly used approaches to quantify and analyze startle potentiation may yield qualitatively different and contradictory conclusions about the effects of focal manipulations or group differences on negative affective response (Grillon & Baas, 2002; Walker & Davis, 2002). In particular, these quantification approaches vary substantially in how they adjust for individual and/or group differences in the influence of activity in the primary startle circuit during neutral conditions. In this report, we review these common approaches to the quantification of startle potentiation in humans and empirically compare them in a simple shock‐threat task.

Neuroscientists (Davis, 2006; Koch, 1999) describe how acoustic startle response magnitude is affected by two neural circuits during shock‐threat tasks in rats: a primary, obligatory circuit and a secondary, modulatory circuit. The obligatory circuit consists of a simple neural pathway from the cochlear root neurons through the nucleus reticularis pontis caudalis to the spinal cord (whole body startle) or facial motor nucleus (pinna reflex). This obligatory circuit is engaged by the startle probe, which is a reflex‐eliciting stimulus that is intense and has a rapid rise time. The modulatory circuit involves both direct and indirect projections from the central nucleus of the amygdala to the reticularis pontis caudalis. This modulatory circuit potentiates the startle response when elicited in the presence of a threatening stimulus that predicts an aversive outcome (e.g., threat cue that signals electric shock) relative to a neutral, nonthreatening stimulus (e.g., no‐threat cue). However, the functional form (e.g., additive, multiplicative) of this modulatory input to the primary, obligatory circuit has not yet been precisely determined. Therefore, multiple approaches have been proposed to quantify startle potentiation due to the many possible forms of this modulatory input. These approaches differ in their explicit or implicit assumptions about how best to adjust potentiation scores across individuals, groups, and experimental conditions that may differ with respect to the level of activity in the primary, obligatory circuit.

The three most commonly used approaches to quantify startle potentiation are based on (1) raw potentiation, (2) standardized potentiation, or (3) percent‐change potentiation. In the first raw potentiation approach, startle potentiation is quantified as the difference between raw (i.e., untransformed) startle response in the threat versus no‐threat conditions. These raw potentiation scores then serve as the dependent measure in an analysis of variance general linear model (ANOVA/GLM) that includes the other focal manipulations or group IVs.^1^ In contrast to the next two approaches we describe, no adjustment is applied to the magnitude of these raw difference scores. However, it should be noted that the magnitude of raw potentiation is typically greater for participants with higher startle response in neutral conditions (Bradford, Kaye, & Curtin, 2014). Use of raw potentiation scores has been our laboratory's longstanding, preferred approach (e.g., Baskin‐Sommers, Curtin, & Newman, 2011; Bradford, Shapiro, & Curtin, 2013; Curtin, Lang, Patrick, & Stritzke, 1998; Curtin, Patrick, Lang, Cacioppo, & Birbaumer, 2001; Hogle & Curtin, 2006; Moberg & Curtin, 2009).

In the second standardized potentiation approach, the raw startle response is first standardized at the level of individual trials using a within‐subject *T* score (or statistically equivalent *z* score) transformation (e.g., Yancey, Vaidyanathan, & Patrick, 2014). This yields transformed trial‐level startle responses with the same overall mean and standard deviation for all participants. Startle potentiation is then quantified as the difference between the standardized startle responses in the threat versus no‐threat conditions. Subsequent analysis is comparable to the raw potentiation approach described above. Standardization of the startle response is common although numerous subtle differences exist across common standardization methods (e.g., mean response calculated across all trials vs. intertrial interval [ITI]; standard deviation pooled within or across conditions). Recent and classic examples of these standardization methods include Bradley, Codispoti, Cuthbert, and Lang, 2001; Grillon et al., 2015; Levenston, Patrick, Bradley, and Lang, 2000; Nelson et al., 2013; Sege, Bradley, and Lang, 2014; Yancey et al., 2014.

Within‐subject standardization adjusts the size of each participant's potentiation scores by the variability of their raw startle response. This is accomplished by dividing participants’ raw startle responses across trials by the standard deviation of these responses (or a subset of these responses; e.g., from the ITI). This adjustment increases the potentiation scores for participants with low variance while decreasing the potentiation scores of participants with large variance. This adjustment may be useful if the magnitude of participants’ modulatory difference between threat and no‐threat is artifactually dependent on the variance of their responses across individual trials. Nonetheless, raw and standardized startle response approaches will produce comparable results when the size of within‐subject effects is consistent despite individual differences in response variance. The current experiment was not designed to manipulate the relationship between effect size and response variance to explicitly and sensitively contrast raw and standardized approaches to the quantification of startle potentiation. Despite this, we report analyses of the standardized potentiation approach to be complete.

In the third percent‐change potentiation approach, startle potentiation is calculated from the raw startle response as a percent change from the no‐threat to the threat condition: ([raw startle in threat − raw startle in no‐threat]/raw startle in no‐threat)*100. Following this, analysis of percent‐change potentiation proceeds as with the analysis of raw and standardized potentiation described earlier (for examples of the percent‐change potentiation approaches, see Gazendam et al., 2014; Jovanovic et al., 2014; Rich et al., 2005; Vanman, Mejia, Dawson, Schell, & Raine, 2003).

The percent‐change approach adjusts the size of each participant's potentiation scores by the magnitude of their response in the no‐threat condition. This is accomplished by dividing participants’ threat versus no‐threat difference scores by their startle response in the no‐threat condition. This adjustment increases the potentiation scores for participants with low startle response in the no‐threat condition and decreases potentiation scores for participants with high startle response in this condition. This adjustment may be useful if the magnitude of participants’ modulatory difference between threat and no‐threat is artifactually greater when they have higher response in the no‐threat condition. Because of this unique adjustment for magnitude of response in the no‐threat condition, analysis of percent‐change startle potentiation can yield qualitatively different conclusions from the raw and standardized approaches in experiments where focal manipulations (e.g., drug administration; Grillon et al., 2015; Grillon, Sinha, Ameli, & O'Malley, 2000; Rodríguez‐Fornells, Riba, Gironell, Kulisevsky, & Barbanoj, 1999) or groups (e.g., patient groups; see Vaidyanathan et al., 2009, for review) exhibit systematic differences in the magnitude of the startle response in the absence of threat.

Grillon and Baas (2002) raised concerns about the potential for divergent conclusions from the raw versus percent‐change potentiation approaches. Furthermore, they explicitly called for empirical comparisons between these approaches. Later that year, Walker and Davis (2002) compared the raw and percent‐change approaches in rodents across five IVs, which were expected to affect startle response in the no‐threat condition but not necessarily startle potentiation. Although they report a preference for percent change, the basis for this conclusion is equivocal. Two of the IVs in these experiments did not clearly support either approach (i.e., startle probe intensity; corticotropin releasing hormone [CRH] administration). Two of the IVs are difficult to interpret because the rodents’ fear response may have been expected to covary with the IV (i.e., participants grouped by general startle reactivity; strychnine administration). For the final IV (i.e., unsignaled footshock), potentiation was more stable when measured by raw potentiation than percent change. Furthermore, it can be argued that guidelines for quantification/analysis of startle potentiation in humans may be more confidently established from experiments with humans given differences in measurement across species (e.g., full body startle vs. eyeblink electromyography) that may affect the measures’ respective statistical properties (e.g., floor/ceiling effects).

In this report, we compare startle potentiation quantification approaches in a simple shock‐threat task in humans with three experimental manipulations (probe intensity, time, and alcohol administration) that have well‐established, robust effects on startle response in no‐threat conditions. Given their effects on startle response, these manipulations should produce a divergent pattern of results for the percent change relative to the raw and standardized approaches. Critically, we also chose these three manipulations because they afforded clear (and verifiable) assertions for their expected effects on participants’ fear of the shock threat (i.e., stable fear across probe intensity and time, reduced fear following alcohol administration). In addition to these three manipulations, we measured general startle reactivity (measured as baseline startle reactivity in this study) to allow us to explore the relationship between individual differences in general startle reactivity and startle potentiation across the three quantification approaches. We collected self‐reported fear/anxiety of the shock threat to further substantiate our predictions regarding the expected effects of each of these IVs in the shock‐threat task. We propose that the following pattern of IV effects should be observed given a valid quantification of startle potentiation:

1. Intensity (95 vs. 100 vs. 105 dB). Startle response varies systematically with the intensity of the eliciting startle probe (Blumenthal & Berg, 1986; Cuthbert, Bradley, & Lang, 1996). A valid approach to the quantification of startle potentiation should yield stable potentiation scores across probe intensities despite this increase in startle response. This assertion follows from two basic assumptions. First, participants’ fear following presentation of a shock‐threat cue should not vary based on the independent intensity of the subsequent startle probe. Second, contemporary neuroscience suggests that the startle probe is processed by and impacts the obligatory but not modulatory circuit of the startle response (Bradley, Lang, & Cuthbert, 1993; Davis & File, 1984; Walker & Davis, 2002).
2. Time (first vs. second half of the experiment). Startle response habituates over repeated probe trials across the first and second half of experiments (Bradley et al., 1993). A valid approach to the quantification of startle potentiation should yield stable scores across the experiment despite this reduction in the startle response. This assertion follows from early validation studies that observed habitation in the obligatory but not modulatory startle response circuits (Bradley et al., 1993; Campeau, Liang, & Davis, 1990; Davis & File, 1984). Of course, it remains possible that the inputs to the modulatory startle response circuit could vary over time. Therefore, we confirm that participants’ fear/anxiety response to shock threat was stable across this experiment via self‐report.
3. Alcohol (no‐alcohol versus alcohol). Startle response is reduced robustly by alcohol administration (Grillon et al., 2000; Stritzke, Patrick, & Lang, 1995). Despite this reduction in startle response, a valid approach to the quantification of startle potentiation should remain sensitive to the well‐documented stress response dampening (SRD) properties of this anxiolytic drug (Sher, 1987). Specifically, alcohol has been demonstrated to reduce behavioral, subjective, and physiological indicators of fear/anxiety and to diminish amygdala response to threat using fMRI (Armeli et al., 2003; Bartholow, Henry, Lust, Saults, & Wood, 2011; Levenson, Sher, Grossman, Newman, & Newlin, 1980; Sher, Bartholow, Peuser, Erickson, & Wood, 2007; Sripada, Angstadt, McNamara, King, & Phan, 2011). We also confirm that alcohol reduced participants’ fear/anxiety to shock threat in this experiment via self‐report.
4. General startle reactivity. Startle response in experimental tasks is strongly positively related to general startle reactivity measured during a baseline procedure (Bradford, Kaye, & Curtin, 2014). We do not offer a precise a priori specification regarding the appropriate relationship between general startle reactivity and startle potentiation during shock threat given valid quantification of startle potentiation. However, recent theory and empirical evidence suggests that general startle reactivity may index individual differences in defensive reactivity to aversive stimuli generally (Bradford, Kaye, & Curtin, 2014; Vaidyanathan et al., 2009). If true, general startle reactivity should be positively related to startle potentiation in the shock‐threat task. Alternatively, if individual differences in general startle reactivity indexes sources of variance that are independent of affect and/or defensive response, general startle reactivity and startle potentiation during shock threat should be unrelated. We explore the relationship between general startle reactivity and fear/anxiety to shock threat via self‐report.

In addition to evaluating the stability and sensitivity of the three startle potentiation quantification approaches across these four IVs, we also conduct supplemental analyses of the reliability (split‐half internal consistency) and validity (criterion correlations with self‐report) for each approach.

## Method

### Participants

We recruited 96 participants (49 female; mean age = 22.1 years, *SD* = 2.0 years) from the university community. Participants were at least 21 years old, had experience within the last year with the study dose of alcohol, reported no history of alcohol‐related problems, no current psychiatric medication use, no alcohol contraindicated medical condition, and were not pregnant (verified by urine sample). We paid participants $10/h or class extra‐credit points for their participation.

### General Startle Reactivity Assessment

Stimulus presentation was controlled by a PC‐based MATLAB script using the Psychophysics Toolbox (Brainard, 1997; Pelli, 1997). Prior to beverage assignment, we measured participants’ general startle reactivity in a baseline procedure (see Startle Response Measurement below). During this assessment, participants viewed a series of yellow and blue colored squares with a diagonal of approximately 7.5 in. presented in the center of a CRT monitor for 5 s each with a 14‐s ITI. No shocks were administered during this assessment.

### Beverage Manipulation

We randomly assigned participants initially to one of three beverage conditions from the standard balanced placebo design (Rohsenow & Marlatt, 1981): alcohol (*N* = 48), no‐alcohol/told alcohol (i.e., placebo; *N* = 24), and no‐alcohol/told no‐alcohol (*N* = 24). We informed participants in the alcohol and no‐alcohol/told alcohol (placebo) conditions that they would receive a moderately impairing dose of alcohol that should produce a blood alcohol concentration (BAC) of approximately 0.08%.The alcoholic beverage consisted of 100‐proof vodka (Smirnoff Blue Label) and a juice mixer, with the juice accounting for three quarters of the drink volume. We calculated the alcohol dose to produce a target BAC of 0.08% approximately 30 min after beverage consumption (see Curtin & Fairchild, 2003, for details regarding the dosing formula). Participants assigned to the no‐alcohol/told alcohol (placebo) condition received a beverage consisting of fruit juice mixed with water poured from a vodka bottle in their presence. Outside of participants’ view, beverages in the alcohol and no‐alcohol/told alcohol (placebo) conditions were misted with alcohol, and 2 ml of alcohol was floated on top of the beverages to provide sensory stimulation to support the placebo manipulation. Participants in the no‐alcohol/told no‐alcohol condition simply drank juice mixer matched to the total drink volume of the beverages in the other two conditions. We divided beverages in all three conditions into two drinks, each consumed over 15 min, for a total drinking period of 30 min. The experimental session began 15 min after the end of the drinking period. We measured BAC via breathalyzer (Alcosensor IV; Intoximeters Inc., St. Louis, MO) immediately before, at the midpoint of, and immediately after completion of the main shock‐threat task.

The use of separate no‐alcohol/told alcohol (placebo) and no‐alcohol/told no‐alcohol conditions from the balanced placebo design is common in alcohol administration research to rule out the possibility of alcohol expectancy effects. If expectancy effects are not observed, these two no‐alcohol conditions can be combined to provide equal *N* alcohol and no‐alcohol conditions. Preliminary analyses coded two regressors to test contrasts among the three beverage conditions in this experiment. However, no significant differences were detected between the two no‐alcohol conditions for the primary dependent variables. Therefore, we combined these two no‐alcohol conditions and proceeded with a single beverage condition regressor that contrasted alcohol (*N* = 48) versus no‐alcohol (*N* = 48) in the final analyses (see Data Analysis Strategy below).

### Shock Tolerance Assessment

Five minutes after the drinking period, we measured participants’ subjective shock tolerance to a series of 200‐ms electric shocks of increasing intensity (7 mA maximum) using standard procedures (Curtin et al., 2001). We administered electric shocks using a custom shock stimulator (Bradford, Magruder, Korhumel, & Curtin, 2014) via stainless steel electrodes across the distal phalanges of the index and ring fingers of the left hand. The procedure was stopped once participants reached the maximum level of shock that they could tolerate. We set shock intensity during the main task to each participant's subjective maximum tolerance threshold to minimize individual differences in shock tolerance.

### Shock‐Threat Task

Participants viewed a series of 84 shock‐threat and no‐threat square cues (equal‐probable) presented in color on a CRT monitor for 5 s each separated by a variable ITI (10–14 s, mean = 12 s). Cues were either blue or yellow in color (counterbalanced for shock and no‐shock across participants). The diagonal of the cues measured approximately 7.5 in. Cues were positioned in the center of the computer monitor. We instructed participants that shocks would be administered during the majority of the threat cue presentations and not during presentation of the no‐threat cues or ITIs. Shocks occurred 4.8 s after cue onset. Actual shock contingency for threat cues was 50%. We measured self‐reported fear/anxiety of the shock threat (1 = *not at all fearful/anxious*; 7 = *extremely fearful/anxious*) at midpoint and task completion.

### Startle Response Measurement

We placed two 4‐mm Ag‐AgCl sensors (TDE‐023; Discount Disposables, St. Albans, VT) filled with conductive gel (ECI Electro‐Gel; Electro‐cap International, Eaton, OH) over the orbicularis oculi muscle under the right eye according to published guidelines (Blumenthal et al., 2005; Bradford, Magruder et al., 2014). We used a NeuroScan Synamps2 bioamplifier (Compumedics Neuroscan, Charlotte, NC) to sample (2500 Hz; 24‐bit A‐D conversion), amplify (DC gain = 10×; AC gain = 2010×), and band‐pass filter (1–500 Hz) the electromyographic signal.

We measured eyeblink startle response to 50‐ms white noise probes with near instantaneous rise time. All noise probes were 100 dB during the baseline procedure. We manipulated noise probe intensity across three levels (95, 100, and 105 dB) during the shock‐threat task. We presented six noise probes at 4.5 s postcue onset during the baseline procedure. We presented 48 noise probes (24 each for threat and no‐threat) at 4.5 s postcue onset during the shock‐threat task. We presented an additional 24 noise probes (eight per probe intensity) during the ITIs in the shock‐threat task to decrease probe predictability. We matched serial position of probes across probe intensity and cue types (threat vs. no‐threat) within participants in two counterbalanced orders. We also presented three habituation probes at the start of the baseline and shock‐threat tasks that were not included in any analyses. A minimum of 14.5 s separated each probe from any previous startle‐eliciting event (i.e., another probe or shock).

We conducted offline data processing using the PhysBox plugin (Curtin, 2011) within the EEGLAB toolbox (Delorme & Makeig, 2004) in MATLAB (The Math Works Inc., Natick, MA). We followed published guidelines for startle response reduction and processing (Blumenthal et al., 2005; Bradford, Magruder et al., 2014). Specifically, we high‐pass filtered (4th order 28 Hz Butterworth filter), epoched (−50–250 ms surrounding probe), rectified, and smoothed (4th order 30 Hz Butterworth low‐pass filter) the data. We rejected trials with greater than ± 20 μV deflections in the 50‐ms preprobe baseline as artifact (i.e., unstable baseline). We scored peak eyeblink startle response between 20 and 100 ms postprobe onset relative to mean 50‐ms preprobe baseline.^2^

We calculated general startle reactivity as the average startle response to the six probes during cues in the baseline assessment. We calculated startle potentiation in the shock‐threat task for the raw potentiation approach as the difference between raw startle responses during threat versus no‐threat cues. We calculated startle potentiation for the standardized approach as the difference between standardized startle responses during threat versus no‐threat cues following within‐subject *T* score standardization^3^of the raw startle response. We calculated startle potentiation for the percent‐change approach from raw startle responses during threat and no‐threat cues as ([threat − no‐threat]/no‐threat)*100.

### Open Science Practices

We support emerging open science guidelines (Nosek et al., 2015). Following these guidelines, we have made the data and analysis scripts associated with this report publicly available via Open Science Framework. These materials can be accessed at osf.io/5nfvu

## Results

### Manipulation Checks

Participants in the alcohol condition achieved a mean BAC of 0.08% (*SD* = 0.01) consistent with our planned target BAC of 0.08%. On average, participants achieved their shock tolerance threshold on shock number 14.8 (*SD* = 6.0; range = 1–25) in the shock tolerance assessment. There was no significant difference in shock tolerance thresholds between the alcohol (*M* = 14.9, *SD* = 6.0, range = 4–25) and no‐alcohol conditions (*M* = 14.6, *SD* = 6.1, range = 1–25), *t*(94) = 0.20, *p* = .839.

Table 1 presents cell and marginal means for raw startle response by condition (ITI, no‐threat, and shock‐threat), probe intensity (95, 100, and 105 dB), time (first half and second half), and beverage condition (no‐alcohol and alcohol). We confirmed that raw startle response was significantly greater during shock threat (*M* = 76.7, *SD* = 79.4) than no‐threat cues (*M* = 46.4, *SD* = 59.4), *t*(93) = 9.24, *p* < .001, demonstrating that our threat of shock manipulation was successful. We also confirmed that all four of our primary IVs were significantly related to raw startle response magnitude as expected. Specifically, raw startle response increased significantly with increasing probe intensity, 95 dB (*M* = 53.1, *SD* = 63.5), 100 dB (*M* = 62.4, *SD* = 69.3), 105 dB (*M* = 69.2, *SD* = 73.2); *F*(2,186) = 51.89, *p* < .001. Pairwise contrasts confirmed significant mean differences in raw startle response across all three probe intensities (*p*s < .001). Raw startle response decreased significantly over time from the first half (*M* = 70.3, *SD* = 74.9) to the second half (*M* = 52.8, *SD* = 62.4) of the experiment; *t*(93) = 8.44, *p* < .001. Raw startle response was significantly lower in the alcohol (*M* = 28.2, *SD* = 24.3) than the no‐alcohol (*M* = 95.0, *SD* = 80.8) beverage condition, *t*(92) = 5.42, *p* < .001. Raw startle response was significantly correlated with general startle reactivity, *r*(92) = .81, *p* < .001.

**Table 1:** Cell and Marginal Means/Standard Deviations for Raw Startle Response by Probe Intensity, Time, and Beverage Condition

First Half of Experiment																
	Probe intensity															
	Low				Medium				High				All probes			
Beverage group	ITI	No‐threat	Shock‐threat	Overall cue	ITI	No‐threat	Shock‐threat	Overall cue	ITI	No‐threat	Shock‐threat	Overall cue	ITI	No‐threat	Shock‐threat	Overall cue
Alcohol	17.6 (15.0)	18.1 (20.8)	39.1 (40.1)	28.6 (27.2)	25.7 (26.8)	19.7 (19.8)	48.8 (52.1)	34.2 (32.6)	30.2 (29.8)	29.0 (30.7)	50.2 (45.2)	39.6 (36.2)	24.5 (22.4)	22.3 (22.1)	46.0 (42.7)	34.2 (30.5)
No‐alcohol	82.6 (87.3)	76.7 (86.8)	111.5 (92.3)	94.1 (87.5)	94.8 (89.0)	85.6 (82.3)	131.0 (98.7)	108.3 (88.6)	102.1 (93.9)	97.7 (85.6)	136.3 (98.6)	117.0 (90.0)	93.2 (88.3)	86.7 (83.4)	126.3 (95.2)	106.5 (87.9)
All participants	50.1 (70.3)	47.4 (69.3)	75.3 (79.6)	61.4 (72.4)	60.3 (74.0)	52.7 (68.1)	89.9 (88.7)	71.3 (76.1)	66.1 (78.1)	63.4 (72.7)	93.2 (87.7)	78.3 (78.5)	58.8 (72.8)	54.5 (68.8)	86.1 (83.7)	70.3 (74.9)

Second Half of Experiment																
	Probe intensity															
	Low				Medium				High				All probes			
Beverage group	ITI	No‐threat	Shock‐threat	Overall cue	ITI	No‐threat	Shock‐threat	Overall cue	ITI	No‐threat	Shock‐threat	Overall cue	ITI	No‐threat	Shock‐threat	Overall cue
Alcohol	13.1 (13.6)	12.4 (11.9)	24.6 (22.6)	18.5 (16.3)	15.6 (16.9)	15.2 (14.9)	28.1 (24.0)	21.6 (18.2)	19.9 (21.4)	18.7 (20.9)	34.6 (33.0)	26.6 (24.9)	16.2 (16.7)	15.4 (15.0)	29.1 (25.3)	22.3 (19.2)
No‐alcohol	61.6 (81.0)	48.5 (52.8)	94.0 (87.3)	71.3 (68.0)	65.8 (74.6)	62.8 (61.9)	108.3 (97.4)	85.6 (76.4)	79.2 (81.7)	73.0 (75.6)	113.9 (93.9)	93.5 (82.0)	68.9 (77.7)	61.4 (61.6)	105.4 (91.4)	83.4 (74.8)
All participants	37.4 (62.7)	30.5 (42.2)	59.3 (72.4)	44.9 (55.9	40.7 (59.4)	39.0 (50.8)	68.2 (81.3)	53.6 (63.9)	49.5 (66.4)	45.8 (61.5)	74.3 (80.6)	60.0 (69.0)	42.5 (61.9)	38.4 (50.2)	67.3 (76.9)	52.8 (62.4)

Full Experiment																
	Probe intensity															
	Low				Medium				High				All probes			
Beverage group	ITI	No‐threat	Shock‐threat	Overall cue	ITI	No‐threat	Shock‐threat	Overall cue	ITI	No‐threat	Shock‐threat	Overall cue	ITI	No‐threat	Shock‐threat	Overall cue
Alcohol	15.4 (13.6)	15.2 (15.0)	31.9 (30.2)	23.6 (21.0)	20.7 (21.0)	17.4 (16.8)	38.4 (35.5)	27.9 (24.0)	25.1 (25.0)	23.8 (24.7)	42.4 (38.1)	33.1 (30.1)	20.4 (19.2)	18.8 (17.9)	37.6 (33.2)	28.2 (24.3)
No‐alcohol	72.1 (83.0)	62.6 (68.8)	102.8 (88.5)	82.7 (76.9)	80.3 (80.9)	74.2 (70.9)	119.7 (96.4)	96.9 (81.9)	90.6 (86.1)	85.3 (79.3)	125.1 (94.9)	105.2 (85.2)	81.0 (82.4)	74.1 (72.0)	115.8 (92.3)	95.0 (80.8)
All participants	43.7 (65.7)	38.9 (54.9)	67.3 (75.0)	53.1 (63.5)	50.5 (66.0)	45.8 (58.6)	79.1 (83.0)	62.4 (69.3)	57.8 (71.2)	54.6 (66.1)	83.8 (83.1)	69.2 (73.2)	50.7 (66.8)	46.44 (59.1)	76.7 (79.4)	61.6 (68.2)

We also confirmed the expected effects of time, beverage condition, and general startle reactivity on self‐reported fear/anxiety during the shock‐threat task.^4^Specifically, fear/anxiety did not change significantly across the experiment (first half: *M* = 3.6, *SD* = 1.5; second half: *M* = 3.3, *SD* = 1.5), *t*(65) = 1.89, *p* = .063. Fear/anxiety was significantly lower in the alcohol (*M* = 3.0, *SD* = 1.3) versus the no‐alcohol beverage condition (*M* = 3.9, *SD* = 1.3), *t*(69) = 3.06, *p* = .003. General startle reactivity measured during the baseline procedure was significantly positively correlated with fear/anxiety during the shock‐threat task, *r*(67) = .25, *p* = .038.

### Primary Comparison of Startle Potentiation Quantification Approaches

We analyzed raw potentiation, standardized potentiation, and percent‐change potentiation in separate GLMs using R (R Development Core Team, 2014).^5^, ^6^ We included additive effects to model repeated measures for probe intensity (95 vs. 100 vs. 105 dB) and time (first vs. second half). We also included additive effects for between‐subjects regressors for beverage condition (no‐alcohol vs. alcohol) and general startle reactivity (measured quantitatively and mean centered) in all models. In all analyses, we coded the beverage condition regressor such that within‐subject effects (i.e., probe intensity, time) were evaluated in the no‐alcohol condition. We followed up significant omnibus effects of probe intensity with three planned pairwise contrasts using Fisher's LSD (least significant difference) approach to protect against inflation of familywise error (Kirk, 1995). We report both GLM coefficients (*b*) and partial eta‐squared (η_p_^2^) to describe effect sizes.

#### Probe intensity

**Figure 1:**
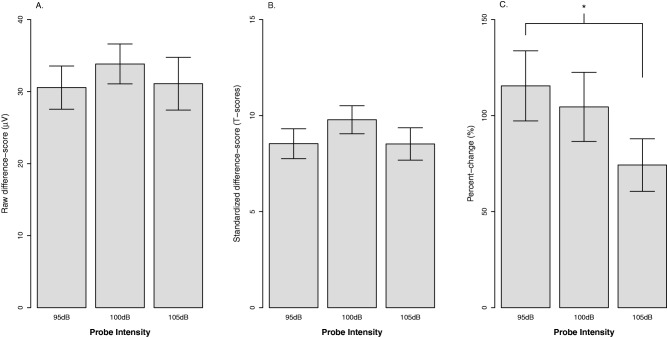
The effect of probe intensity on startle potentiation by quantification approach. A: Raw potentiation approach. B: Standardized potentiation approach. C: Percent‐change potentiation approach. Confidence bars represent ± one standard error for point estimates of startle potentiation from the GLM. *p < .05.

Figure 1 displays the effect of probe intensity for each quantification approach. We proposed that a valid approach for startle potentiation should be stable across probe intensities used to elicit and measure the response.^7^ Consistent with this, the effect of probe intensity was not significant for either raw potentiation, *F*(2,166) = .79, *p* = .457, η_p_^2^ = .01, or standardized potentiation, *F*(2,182) = 1.54, *p* = .217, η_p_^2^ = .02. In contrast, percent‐change potentiation was not stable across probe intensities, *F*(2,172) = 3.51, *p* = .032, η_p_^2^ = .04. Percent‐change potentiation decreased with increasing probe intensity, and pairwise contrasts indicated a significant difference between 95 and 105 dB, *b* = −41.3, *t*(86) = 2.32, *p* = .023, η_p_^2^ = .06.

#### Time

**Figure 2:**
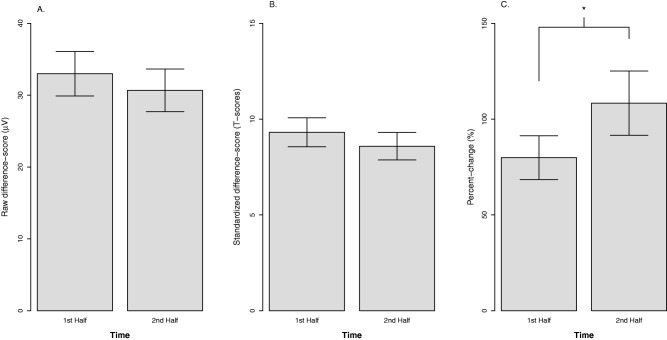
The effect of time on startle potentiation by quantification approach. A: Raw potentiation approach. B: Standardized potentiation approach. C: Percent‐change potentiation approach. Confidence bars represent ± one standard error for point estimates of startle potentiation from the GLM. *p < .05.

Figure 2 displays the effect of time (first vs. second half of experiment) for each quantification approach. We expected stable negative affective response to the shock threat across time. As reported earlier, self‐reported fear/anxiety did not significantly change across time in this experiment. Consistent with self‐report, neither raw potentiation, *b* = −2.3, *t*(83) = .86, *p* = .392, η_p_^2^ = .01, nor standardized potentiation, *b* = ‐.73, *t*(91) = .93, *p* = .355, η_p_^2^ = .01, changed significantly across time. In contrast, percent‐change potentiation increased significantly over time, *b* = 28.4, *t*(86) = 2.21, *p* = .030, η_p_^2^ = .05.

#### Beverage condition

**Figure 3:**
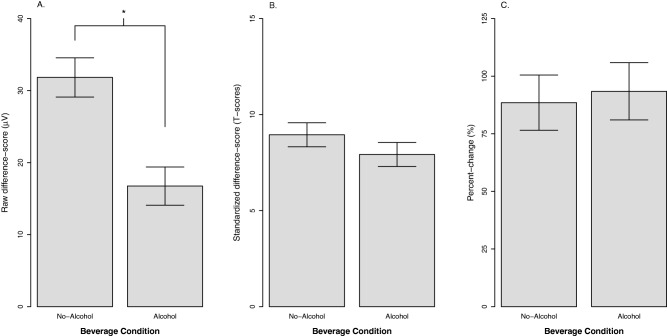
The effect of beverage condition on startle potentiation by quantification approach. A: Raw potentiation approach. B: Standardized potentiation approach. C: Percent‐change startle potentiation approach. Confidence bars represent ± one standard error for point estimates of startle potentiation from the GLM. *p < .05.

Figure 3 displays the effect of beverage condition (no‐alcohol vs. alcohol) for each quantification approach. We expected that alcohol would significantly reduce negative affect based on the large literature documenting its anxiolytic, stress response‐dampening properties. In addition, alcohol reduced self‐reported fear/anxiety in this experiment as reported earlier. Consistent with our prediction and self‐report results, alcohol significantly reduced raw potentiation, *b* = −15.1, *t*(83) = 3.91, *p* < .001, η_p_^2^ = .16. In contrast, alcohol did not significantly change either standardized potentiation, *b* = −1.02, *t*(91) = 1.15, *p* = .254, η_p_^2^ = .01, or percent‐change potentiation, *b* = 4.9, *t*(86) = .28, *p* = .779, η_p_^2^ = .00.

#### General startle reactivity

**Figure 4:**
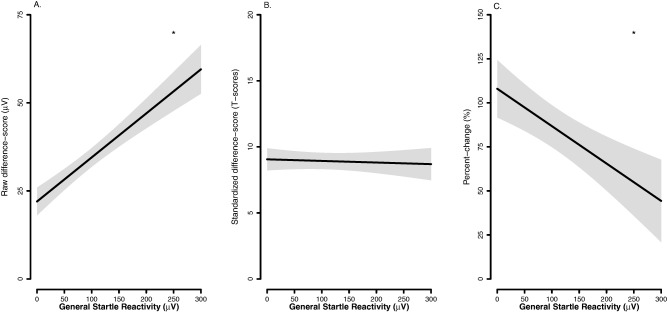
The effect of general startle reactivity on startle potentiation by quantification approach. A: Raw potentiation approach. B: Standardized potentiation approach. C: Percent‐change potentiation approach. Lines and shaded confidence bands represent mean predicted startle potentiation and ± one standard error for point estimates, respectively, of startle potentiation from the GLM. *p < .05.

Figure 4 displays the relationship between general startle reactivity and startle potentiation for each quantification approach. We did not offer strong a priori predictions regarding the expected relationship between general startle reactivity and startle potentiation, although we suggested that either a positive or no relationship could be supported by existing theory and/or empirical evidence. As reported earlier, a significant positive relationship was observed between general startle reactivity and self‐reported fear/anxiety in this experiment. Consistent with self‐report, general startle reactivity and raw potentiation were significantly positively related, *b* = 0.1, *t*(83) = 4.03, *p* < .001, η_p_^2^ = .16. The relationship between general startle reactivity and standardized potentiation was not significant, *b* = 0.0, *t*(91) = .22, *p* = .823, η_p_^2^ = .00. General startle reactivity and percent‐change potentiation were significantly negatively related, *b* = −0.2, *t*(86) = 2.03, *p* = .045, η_p_^2^ = .05.

### Supplemental Analyses of Reliability and Validity

We tested correlations between potentiation scores derived separately from odd and even trials to assess the internal consistency reliability of the three approaches. These correlations were significant for raw potentiation (*r* = .81; *df* = 92; *p* < .001), standardized potentiation (*r* = .53; *df* = 92; *p* < .001), and percent‐change potentiation (*r* = .72; *df* = 92; *p* < .001). Pairwise tests of differences between these correlations indicated that the correlation for the standardized approach was significantly lower than that observed for both raw potentiation (*z* = 3.62 *p* < .001) and percent change approaches (*z* = 2.14, *p* = .032). The correlations yield Spearman‐Brown corrected internal consistency reliability estimates of 0.90 for raw potentiation, 0.69 for standardized potentiation, and 0.84 for percent‐change potentiation for the full measures using all trials.

We conducted supplemental validity analyses by testing correlations of self‐reported fear/anxiety during the shock‐threat task with each startle potentiation quantification approach. The correlations between self‐reported fear/anxiety and startle potentiation were significant for both raw (*r* = .38, *df* = 67, *p* = .001) and standardized potentiation (*r* = .34, *df* = 67, *p* = .005). The correlation with percent‐change potentiation was nonsignificant (*r* = .17, *df* = 67, *p* = .160). Tests of differences between correlations were not significant for contrasts among these three approaches (*p*s > .088).

## Discussion

Our four IVs—probe intensity, time, alcohol administration, and general startle reactivity—all affected startle response in the shock‐threat task. These effects provide an experimental context where the three quantification approaches could yield different conclusions regarding the effects of our four IVs on startle potentiation. Analyses of participants’ self‐report confirmed that fear/anxiety of the shock threat did not change over the course of the experiment, was reduced by alcohol, and was positively related to general startle reactivity at baseline. These analyses substantiated our a priori assertions regarding the appropriate IV effects on startle potentiation given a valid quantification approach for startle potentiation. With the validity of our test bed established by these manipulation checks, we were well positioned to offer recommendations regarding the quantification of startle potentiation and to identify issues that warrant further examination.

### The Percent‐Change Approach

Our analyses generate substantial concerns about the validity and sensitivity of the percent‐change approach. First, percent‐change potentiation significantly decreased with increasing probe intensity. If valid, we would have to conclude that participants’ fear/anxiety of the shock‐threat phasically decreased when their startle reflex was elicited by more intense probes, even though probe intensity was unpredictable and intermixed within blocks. Unless we conclude that startle response methodology is susceptible to such problematic measurement reactivity based on probe intensity, this instability associated with percent‐change substantially undermines its measurement validity.

Second, percent‐change scores significantly increased in the second half of the experiment relative to the first half. This result for percent‐change potentiation is unexpected given the repeated administration of a well‐controlled aversive stimulus (i.e., electric shock) for which stable or possibly habituated response should be expected over time. More directly, the increase in percent‐change potentiation over time was also discordant with participants’ self‐reported fear/anxiety, which marginally but not significantly decreased across time.

Third, percent‐change potentiation failed to detect the expected anxiolytic effect of alcohol. In fact, percent‐change scores were descriptively greater in the alcohol relative to no‐alcohol condition. This conflicts with evidence that suggests that alcohol has anxiolytic, stress response‐dampening properties. Moreover, this result again conflicted with participants’ self‐report, which confirmed the reduction in fear/anxiety by alcohol in this experiment.

Fourth, general startle reactivity at baseline was negatively related to percent‐change potentiation. General startle reactivity may index individual differences in defensive reactivity to aversive stimuli generally (Bradford, Kaye, & Curtin, 2014; Vaidyanathan et al., 2009). Alternatively, individual differences in general startle reactivity may be independent of affect and/or defensive response. Given this, we proposed that a valid approach to the quantification of startle potentiation may yield either a positive or null effect for general startle reactivity. Of note, we observed that general startle reactivity was positively related to self‐reported fear/anxiety of the shock threat in this experiment, which provides further support to expect a positive relationship. Regardless, it is difficult to explain the observed significant negative relationship between general startle reactivity and percent‐change potentiation, which further undermines confidence in the validity of this quantification approach.

Percent change demonstrated adequate reliability, comparable to the raw potentiation approach. However, no significant correlation was observed between percent‐change potentiation and self‐reported fear/anxiety of the shock threat. The aggregate of these findings for the percent‐change approach substantially undermine its validity.

Use of the percent‐change approach for startle potentiation has likely emerged because of longstanding concerns about how the magnitude of responding in neutral conditions influences responses to experimental task stimuli for many psychophysiological measures. This issue has been famously described as the law of initial value (LIV; Wilder, 1967). Wilder's initial formulation of the LIV proposed that “the higher the initial [neutral] value, the smaller the response to function‐raising, and the larger the response to function‐depressing stimuli” (Wilder, 1967, p. viii). However, others subsequently proposed that, when examined appropriately, higher initial values generally lead to increased psychophysiological reactivity except at the upper limits of the measure (Jin, 1992; Myrtek & Foerster, 1986). In fact, empirical tests of the LIV have spurred considerable debate over if, when, how, and for which measures the LIV manifests in psychophysiology, and it may be the exception rather than the rule for most psychophysiological measures (Berntson, Uchino, & Cacioppo, 1994; Furedy & Scher, 1989; Geenen & Van De Vijver, 1993; Jin, 1992; Stern et al., 2001).

The percent‐change approach appears to have emerged to adjust for LIV in the form proposed by Jin and others (Jin, 1992; Myrtek & Foerster, 1986). For startle potentiation, this would be appropriate if the input from the modulatory circuit was positive and multiplicative. If such LIV were present, the adjustment provided by percent change would have yielded stable startle potentiation across changing levels of startle response in the no‐threat condition due to probe intensity and time. This was not the case. Instead, the observed pattern of results from these IVs suggests a functionally additive input from the modulatory startle circuit consistent with the raw and standardized approaches.

### The Raw Versus Standardized Startle Potentiation Approaches

As we acknowledged earlier, the IVs in this experiment were selected to affect startle response in the no‐threat condition. This provided an opportunity to carefully contrast the percent‐change and raw potentiation approaches. Given the nature of the within‐subject standardization transformation, a sensitive contrast of raw and standardized approaches would require direct manipulation of the variance of participants’ responses individually and within specific experimental conditions. Nonetheless, the current experiment afforded us a preliminary opportunity to compare these two approaches. Both raw and standardized potentiation approaches yielded stable startle potentiation scores across both probe intensity and time manipulations. Thus, confirmation of the stability of both approaches across these two IV manipulations provides support for their validity.

The raw potentiation approach was sensitive to the well‐documented anxiolytic effect of alcohol, which we confirmed in this experiment via self‐report (Armeli et al., 2003; Bartholow et al., 2011; Levenson et al., 1980; Sher et al., 2007; Sripada et al., 2011). In contrast, although alcohol descriptively reduced startle potentiation quantified by the standardized approach, this effect was not significant. This putative loss of power for the standardized approach may have resulted from substantial reductions in the variance of raw startle responding across trials for participants in the alcohol condition (e.g., see standard deviations by beverage condition in Table 1). The standardized approach may add noise by amplifying the threat effects of a subset of these participants whose trial variance may be near the floor due to robust reductions of startle magnitude by alcohol. It is possible that the standardized approach may have similar problems with power in experiments more generally when “undetected” nonresponders are included because many of their trials that contain only noise artifact are incorrectly classified as true responses (i.e., false positives) because of conservative methods for the identification of these no‐response trials and/or removal of nonresponders from the sample.

The exploratory analyses of general startle reactivity also produced divergent results across the raw and standardized approaches. General startle reactivity was positively correlated with raw potentiation but uncorrelated with standardized potentiation. If general startle reactivity is unrelated to the strength of participants’ defensive reactivity to threats, then standardized potentiation appears to be superior by removing this artifact. However, general startle reactivity may index real differences across individuals in their propensity to respond to threats. It would not be surprising to us if participants that respond more strongly to the aversive startle probe also respond more strongly to other aversive threats such as shock. If true, the positive correlation between general startle reactivity and raw potentiation may represent valid differences in participants’ responding to threats generally that may be informative and best examined by including general startle reactivity in the analytic model (Bradford, Kaye, & Curtin, 2014). The observed significant relationship between general startle reactivity and self‐reported fear/anxiety of the shock threat in this experiment offers some support for this latter perspective. Of course, more definitive support would require further empirical evidence regarding the psychobiological construct indexed by general startle reactivity.

Our supplemental analyses of reliability provide some support for the raw versus standardized approach. The internal consistency of the raw potentiation approach was the highest of the three approaches (0.90), though only modestly higher than percent change (0.84). However, the internal consistency for the standardized approach was significantly lower overall (0.69) and possibly low enough to impact on statistical power. In contrast, criterion validity correlations with self‐reported fear/anxiety were approximately comparable in magnitude and significant for both raw and standardized potentiation (*r*s = .38 and .34, respectively).

There are other reasons to be cautious about the use of standard scores. As noted in the introduction, there are currently numerous related but distinct methods used within and across laboratories that standardize startle response. Unfortunately, few laboratories publish the specific standardization formula they use, and the ability to choose between standardization formulas may represent an undesirable “researcher degree of freedom” (Simmons, Nelson, & Simonsohn, 2011). In addition, standardizing the effects of IVs based on within‐participant standard deviations that may vary across samples, and/or experimental designs may degrade comparisons across experiments regarding IV effect sizes. Furthermore, the use of raw but not standardized response allows for the presentation of signal‐averaged waveforms if these waveforms are deemed useful to portray effects. Such waveforms are not typical for studies measuring eyeblink startle potentiation but are commonly displayed when the postauricular reflex is measured (e.g., Benning, Patrick, & Lang, 2004).

Fridlund & Cacioppo (1986) described how standardizing responding can sometimes produce unanticipated, suboptimal results. Specifically, they demonstrate that, even if raw response levels across all trials in two conditions are perfectly replicated across experiments, the ordering of the two associated condition means can be artifactually reversed simply by including a third condition that produces more extreme responding. They conclude that standard scores may introduce such problems in any experiment that does not elicit the full range of responding for all participants. Given how difficult this may be to do, they caution against routine and exclusive use of standardized scores (see pp. 583–584 and their Table 1).

Unfortunately, Fridlund and Cacioppo's (1986) early caveats regarding standardization did not motivate sufficient additional direct empirical comparisons of the raw and standardized approaches to yield definitive recommendations regarding these competing approaches. Thus, we strongly call for additional research on the topic that can lead to clear guidelines for the field to follow. When competing approaches exist, it is often considered conservative to recommend that results from both approaches be reported. This recommendation may be appropriate in a measure's infancy but report of conflicting results across quantification approaches does not help advance understanding of the phenomenon of interest and can undermine subsequent use of that measure.

### Other Considerations and Future Directions

We have no reason to believe that our results should not generalize to the quantification of startle potentiation to threats other than shock (e.g., aversive auditory or tactile stimulation; Miller, Curtin, & Patrick, 1999; Schmitz et al., 2011). Our results also may extend to other paradigms that manipulate affective response such as the affective picture‐viewing task (Lang, 1995), but this should be confirmed empirically. Of interest, the percent‐change approach has not been recommended or frequently used for analysis in the picture‐viewing task even when other focal manipulations or groups differ with respect to startle response in the neutral condition. Nonetheless, the empirical evidence presented in this report should further reinforce avoidance of this approach in picture‐viewing tasks. Both raw and standardized potentiation approaches are commonly used in these tasks (for raw, see Baskin‐Sommers, Curtin, & Newman, 2013; Smith, Bradley, & Lang, 2005; Stritzke et al., 1995; for standardized, see Bradley et al., 2001; Levenston et al., 2000; Sege et al., 2014). Research directly contrasting the raw and standardized approaches for the analysis of startle response in the picture‐viewing task is needed given the central role this task plays in affective science.

We believe it is equally important to explicitly acknowledge that our conclusions are unlikely to extend to the use of the startle response to measure processes other than affective response. For example, clear, evidence‐based guidelines currently recommend the use of percent‐change scores for the quantification of startle prepulse inhibition (PPI; Blumenthal et al., 2004). Of course, startle potentiation and PPI are directionally different, thus the impact of the respective denominators on each measure's scores will vary. Perhaps more importantly, startle potentiation and PPI index different psychological constructs (fear/anxiety vs. sensory attentional gating for startle potentiation and PPI, respectively) that have distinct neural modulatory mechanisms (Davis, Walker, Miles, & Grillon, 2010; Hawk & Cook, 2000; Koch, 1999; Swerdlow, Geyer, & Braff, 2001). As such, each likely requires different quantification approaches. Nonetheless, our field needs further direct empirical comparisons and dialogue about quantification for startle potentiation, PPI, and other well‐established psychophysiological measures for which multiple quantification approaches exist.

Alcohol administration provided an attractive pharmacological manipulation to contrast these quantification approaches. Alcohol has both robust effects on startle response in the no‐threat condition, and its effects on fear/anxiety are well established in the literature and confirmed in the current study with self‐report. Nonetheless, future research should examine alternative pharmacological manipulations. In particular, the use of drugs that change startle response magnitude but do not alter affective response would provide an important and necessary extension of the research we report here.

Results from our exploratory analyses of general startle reactivity dovetail attractively with existing research on this potentially interesting individual difference. Vaidyanathan et al. (2009) have suggested that general startle reactivity, measured independent of an affective foreground, may serve as a neurobiological indicator of dispositional defensive reactivity. Vaidyanathan, Malone, Miller, McGue, and Iacono (2014) have recently observed that individual differences in general startle reactivity are highly heritable, which positions it as a potentially attractive endophenotypic marker of defensive reactivity. Furthermore, we have observed that general startle reactivity measured at baseline can identify individuals who will subsequently display exaggerated responding to affective stimuli or more potent effects of drugs and/or drug deprivation (Bradford, Kaye, & Curtin, 2014; Bradford et al., 2013; Gloria, Hefner, Baker, & Curtin, 2015; Hogle, Kaye, & Curtin, 2010). Consistent with these observations, increased general startle reactivity was associated with greater fear/anxiety response to the shock threat when measured either via self‐report or startle potentiation quantified by the raw potentiation approach in this experiment. Given these observations, future research in psychopathology and affective science should more routinely measure general startle reactivity at baseline or otherwise and formally model its effects in subsequent analyses. By anchoring general startle reactivity in a more elaborate nomological network that includes other constructs that we either measure or manipulate, we can refine and clarify this potentially important neurobiological index of fear circuitry consistent with the emerging NIMH RDoC perspective (NIMH–Negative Valence Systems: Workshop Proceedings, 2011). Of course, modeling the effects of general startle reactivity in our analyses, when significant, will further increase our statistical power to test the effects of other focal IVs as well (Bradford, Kaye, & Curtin, 2014).

## Footnotes

1 We explicitly calculate and analyze startle potentiation difference scores for the raw and standardized potentiation approaches to allow us to use identical analysis models across these two approaches and the percent‐change approach. Of course, the results reported from analyses of these within‐subject difference scores are statistically equivalent to the results that would be obtained from analyzing startle response (rather than potentiation difference scores) and including threat as a within‐subject IV.

2 Across all participants (*N* = 96), 1.2% of trials were identified as artifact in the shock‐threat task and rejected prior to subsequent analyses. Across all participants, 4.2% of trials were identified as nonresponse trials, which were defined as trials where peak startle response during the scoring window did not exceed the maximum observed level in the 50‐ms preprobe baseline. These trials were retained in the subsequent analyses. However, two participants in the no‐alcohol condition were identified as startle nonresponders (<5 μV mean general startle reactivity). These two participants were not included in any subsequent analysis of startle response or potentiation.

3 Trial‐level raw startle responses (i) during the no‐shock and shock cues in the main task were standardized within‐subjects using a *T‐*score transformation based on each participant's (j) raw startle response mean (M_j_) and standard deviation (SD_j_) across their 72 trials in the main task (excluding the three habituation trials) using the following formula: TStartle_ij_ = ([RawStartle_ij_ − M_j_)/SD_j_) * 10 + 50

4 We made the decision to include the self‐report measure of fear/anxiety after data collection was initiated, which resulted in *N* = 71 participants available for self‐report analysis (36 alcohol and 35 no‐alcohol participants). We conducted supplemental analyses to confirm that the effects we report for our three quantification approaches (raw startle potentiation, standardized startle potentiation, and percent‐change startle potentiation) for our four primary IVs (probe intensity, time, beverage condition, and general startle reactivity) were comparable across subsamples of participants who did and did not provide self‐report of fear/anxiety. Specifically, we added self‐report data available (yes vs. no) as a factor to our primary analyses for each of the three quantification approaches. This factor did not significantly moderate the effects of any of the four IVs across any of the three approaches, which confirms that the reported effects are comparable in these two subsamples.

5 We conducted case analyses to identify participants who were GLM outliers (i.e., studentized residual with Bonferroni corrected *p* < .05; Fox, 1991) for the primary analyses of the three quantification approaches. These case analyses resulted in the exclusion of eight participants (3 alcohol, 5 no‐alcohol) from analysis for raw potentiation and five participants (4 alcohol, 1 no‐alcohol) from analysis for percent‐change potentiation. No GLM model outliers were identified for standardized potentiation. The pattern of significant/nonsignificant results is identical for raw potentiation with and without these GLM outliers included. The pattern of results for percent change is also identical with one exception. The significant increase in percent‐change potentiation for high versus low probe intensities that is observed with GLM outliers removed (41.3%, *p* = .023) is larger in magnitude but only trend level when GLM outliers are included (47.3%, *p* = .074) due to the larger standard error produced by retaining the model outliers (26.1 vs. 17.8, with and without GLM outliers, respectively).

6 Startle potentiation scores had the following distributional shapes: raw potentiation (skew = 2.2, kurtosis = 6.5), standardized potentiation (skew = 0.2, kurtosis = −0.5), percent‐change potentiation (skew = 3.2, kurtosis = 13.7). We chose to present results in this report without transforming these scores to correct their distributional shape (e.g., positively skewed) because such transformations are uncommon in startle research. Furthermore, distributional transformations can hinder interpretability of psychophysiological data (Stern et al., 2001). However, we performed supplemental analyses of transformed raw and percent‐change potentiation scores (square root and log, respectively) to confirm that our conclusions were robust to this issue. Standardized potentiation scores were already normally distributed in this particular experiment. The results were essentially unchanged for the raw potentiation approach. Results were also consistent for the percent‐change approach except that the effect of time was no longer significant.

7 We used simple additive models for our primary analyses given the focal nature of our study hypotheses. However, we conducted supplemental analyses for each quantification approach that allowed probe intensity to interact with all other IVs. No significant interactions with probe intensity were observed (*p*s > .331), which confirms that the main effects of alcohol, time, and general startle reactivity were consistent across the three probe intensity levels.

## References

[psyp12545-bib-0001] Armeli, S. , Tennen, H. , Todd, M. , Carney, M. A. , Mohr, C. , Affleck, G. , & Hromi, A. (2003). A daily process examination of the stress‐response dampening effects of alcohol consumption. Psychology of Addictive Behaviors, 17, 266–276. doi: 10.1037/0893-164X.17.4.266 14640822 10.1037/0893-164X.17.4.266

[psyp12545-bib-0002] Bartholow, B. D. , Henry, E. A. , Lust, S. A. , Saults, J. S. , & Wood, P. K. (2011). Alcohol effects on performance monitoring and adjustment: Affect modulation and impairment of evaluative cognitive control. Journal of Abnormal Psychology, 121, 173–186. doi: 10.1037/a0023664 21604824 10.1037/a0023664 PMC4254813

[psyp12545-bib-0003] Baskin‐Sommers, A. R. , Curtin, J. J. , & Newman, J. P. (2011). Specifying the attentional selection that moderates the fearlessness of psychopathic offenders. Psychological Science, 22, 226–234. doi: 10.1177/0956797610396227 21245494 10.1177/0956797610396227 PMC3358698

[psyp12545-bib-0004] Baskin‐Sommers, A. R. , Curtin, J. J. , & Newman, J. P. (2013). Emotion‐modulated startle in psychopathy: Clarifying familiar effects. Journal of Abnormal Psychology, 122, 458–468. doi: 10.1037/a0030958 23356218 10.1037/a0030958 PMC3640755

[psyp12545-bib-0005] Benning, S. D. , Patrick, C. J. , & Lang, A. R. (2004). Emotional modulation of the post‐auricular reflex. Psychophysiology, 41, 426–432. doi: 10.1111/j.1469-8986.00160.x 15102128 10.1111/j.1469-8986.00160.x

[psyp12545-bib-0006] Berg, W. K. , & Balaban, M. T. (1999). Startle elicitation: Stimulus parameters, recording techniques, and quantification In Dawson M. E., Schell A. H., & Bohmelt A. H. (Eds.), Startle modification: Implications for neuroscience, cognitive science, and clinical science. Cambridge, UK: Cambridge University Press.

[psyp12545-bib-0007] Berntson, G. G. , Uchino, B. N. , & Cacioppo, J. T. (1994). Origins of baseline variance and the law of initial values. Psychophysiology, 31, 204–210. doi: 10.1111/j.1469-8986.1994.tb01042.x 8153258 10.1111/j.1469-8986.1994.tb01042.x

[psyp12545-bib-0008] Blumenthal, T. D. , & Berg, W. K. (1986). Stimulus rise time, intensity, and bandwidth effects on acoustic startle amplitude and probability. Psychophysiology, 23, 635–641. doi: 10.1111/j.1469-8986.1986.tb00682.x 3823338 10.1111/j.1469-8986.1986.tb00682.x

[psyp12545-bib-0009] Blumenthal, T. D. , Cuthbert, B. N. , Filion, D. L. , Hackley, S. , Lipp, O. V. , & van Boxtel, A. (2005). Committee report: Guidelines for human startle eyeblink electromyographic studies. Psychophysiology, 42, 1–15. doi: 10.1111/j.1469-8986.2005.00271.x 15720576 10.1111/j.1469-8986.2005.00271.x

[psyp12545-bib-0010] Blumenthal, T. D. , Elden, A. , & Flaten, M. A. (2004). A comparison of several methods used to quantify prepulse inhibition of eyeblink responding. Psychophysiology, 41, 326–332. doi: 10.1111/j.1469-8986.2003.00144.x 15032998 10.1111/j.1469-8986.2003.00144.x

[psyp12545-bib-0011] Bradford, D. E. , Kaye, J. T. , & Curtin, J. J. (2014). Not just noise: Individual differences in general startle reactivity predict startle response to uncertain and certain threat. Psychophysiology, 51, 407–411. doi: 10.1111/psyp.12193 24611542 10.1111/psyp.12193 PMC3984356

[psyp12545-bib-0012] Bradford, D. E. , Magruder, K. P. , Korhumel, R. A. , & Curtin, J. J. (2014). Using the threat probability task to assess anxiety and fear during uncertain and certain threat. Journal of Visualized Experiments. Retrieved from http://www.ncbi.nlm.nih.gov/pubmed/25285398 10.3791/51905 PMC4431547 25285398

[psyp12545-bib-0013] Bradford, D. E. , Shapiro, B. L. , & Curtin, J. J. (2013). How bad could it be? Alcohol dampens stress responses to threat of uncertain intensity. Psychological Science, 24, 2541–2549. doi: 10.1177/0956797613499923 24145332 10.1177/0956797613499923 PMC3951286

[psyp12545-bib-0014] Bradley, M. M. , Codispoti, M. , Cuthbert, B. , & Lang, P. J. (2001). Emotion and motivation I: Defensive and appetitive reactions in picture processing. Emotion, 1, 276–298. doi: 10.1037/1528-3542.1.3.276 12934687

[psyp12545-bib-0015] Bradley, M. M. , Lang, P. J. , & Cuthbert, B. N. (1993). Emotion, novelty, and the startle reflex: Habituation in humans. Behavioral Neuroscience, 107, 970–980. doi: 10.1037/0735-7044.107.6.970 8136072 10.1037//0735-7044.107.6.970

[psyp12545-bib-0016] Brainard, D. H. (1997). The psychophysics toolbox. Spatial Vision, 10, 433–436. 9176952

[psyp12545-bib-0017] Campeau, S. , Liang, K. C. , & Davis, M. (1990). Long‐term retention of fear‐potentiated startle following a short training session. Animal Learning & Behavior, 18, 462–468. doi: 10.3758/BF03205328

[psyp12545-bib-0018] Curtin, J. (2011). *PhysBox: The psychophysiology toolbox. An open source toolbox for psychophysiological data reduction within EEGLAB.* Retrieved from http://dionysus.psych.wisc.edu/PhysBox.htm

[psyp12545-bib-0019] Curtin, J. J. , & Fairchild, B. A. (2003). Alcohol and cognitive control: Implications for regulation of behavior during response conflict. Journal of Abnormal Psychology, 112, 424–436. doi: 10.1037/0021-843X.112.3.424 12943021 10.1037/0021-843x.112.3.424

[psyp12545-bib-0020] Curtin, J. J. , Lang, A. R. , Patrick, C. J. , & Stritzke, W. G. K. (1998). Alcohol and fear‐potentiated startle: The role of competing cognitive demands in the stress‐reducing effects of intoxication. Journal of Abnormal Psychology, 107, 547–565. doi: 10.1037/0021-843X.107.4.547 9830242 10.1037//0021-843x.107.4.547

[psyp12545-bib-0021] Curtin, J. J. , Patrick, C. J. , Lang, A. R. , Cacioppo, J. T. , & Birbaumer, N. (2001). Alcohol affects emotion through cognition. Psychological Science, 12, 527–531. doi: 10.1111/1467-9280.00397 11760143 10.1111/1467-9280.00397

[psyp12545-bib-0022] Cuthbert, B. , Bradley, M. M. , & Lang, P. J. (1996). Probing picture perception: Activation and emotion. Psychophysiology, 33, 103–111. doi: 10.1111/j.1469-8986.1996.tb02114.x 8851238 10.1111/j.1469-8986.1996.tb02114.x

[psyp12545-bib-0023] Davis, M. (2006). Neural systems involved in fear and anxiety measured with fear‐potentiated startle. American Psychologist, 61, 741–756. doi: 10.1037/0003-066X.61.8.741 17115806 10.1037/0003-066X.61.8.741

[psyp12545-bib-0024] Davis, M. , & File, S. E. (1984). Intrinsic and extrinsic mechanisms of habituation and sensitization: Implications for the design and analysis of experiments In Peeke H. V. S. & Petrinovich L. (Eds.), Habituation, sensitization, and behavior (pp. 287–323). Waltham, MA: Academic Press Retrieved from http://www.sciencedirect.com/science/article/pii/B9780125498609500158

[psyp12545-bib-0025] Davis, M. , Walker, D. L. , Miles, L. , & Grillon, C. (2010). Phasic vs sustained fear in rats and humans: Role of the extended amygdala in fear vs anxiety. Neuropsychopharmacology Reviews, 35, 105–135. doi: 10.1037/npp.2009 19693004 10.1038/npp.2009.109 PMC2795099

[psyp12545-bib-0026] Delorme, A. , & Makeig, S. (2004). EEGLAB: An open source toolbox for analysis of single‐trial EEG dynamics including independent component analysis. Journal of Neuroscience Methods, 134, 9–21. doi: 10.1016/j.jneumeth.2003.10.009 15102499 10.1016/j.jneumeth.2003.10.009

[psyp12545-bib-0027] Fox, J. (1991). Regression diagnostics (Vol. 07–079). Iowa City, IA: Sage Publications Inc.

[psyp12545-bib-0028] Fridlund, A. J. , & Cacioppo, J. T. (1986). Guidelines for human electromyographic research. Psychophysiology, 23, 567–589. doi: 10.1111/j.1469-8986.1986.tb00676.x 3809364 10.1111/j.1469-8986.1986.tb00676.x

[psyp12545-bib-0029] Furedy, J. J. , & Scher, H. (1989). The law of initial values: Differentiated Testing as an empirical generalization versus enshrinement as a methodological rule. Psychophysiology, 26, 120–122. doi: 10.1111/j.1469-8986.1989.tb03140.x 2922452 10.1111/j.1469-8986.1989.tb03140.x

[psyp12545-bib-0030] Gazendam, F. J. , Kamphuis, J. H. , Eigenhuis, A. , Huizenga, H. M. H. , Soeter, M. , Bos, M. G. N. , … Kindt, M. (2014). Personality predicts individual variation in fear learning: A multilevel growth modeling approach. Clinical Psychological Science, 2167702614535914. doi: 10.1177/2167702614535914

[psyp12545-bib-0031] Geenen, R. , & Van De Vijver, F. J. R. (1993). A simple test of the law of initial values. Psychophysiology, 30, 525–530. doi: 10.1111/j.1469-8986.1993.tb02076.x 8416079 10.1111/j.1469-8986.1993.tb02076.x

[psyp12545-bib-0032] Gloria, R. , Hefner, K. R. , Baker, T. , & Curtin, J. (2015). *Uncovering a potential biological marker for marijuana withdrawal: Startle potentiation to threat*. Manuscript in preparation.

[psyp12545-bib-0033] Grillon, C. , & Baas, J. M. (2002). Comments on the use of the startle reflex in psychopharmacological challenges: Impact of baseline startle on measurement of fear‐potentiated startle. Psychopharmacology, 164, 236–238. 12481758 10.1007/s00213-002-1164-5

[psyp12545-bib-0034] Grillon, C. , & Baas, J. M. (2003). A review of the modulation of the startle reflex by affective states and its application in psychiatry. Clinical Neurophysiology, 144, 1557–1579. doi: 10.1016/S1388-2457(03)00202-5 12948786 10.1016/s1388-2457(03)00202-5

[psyp12545-bib-0035] Grillon, C. , Hale, E. , Lieberman, L. , Davis, A. , Pine, D. S. , & Ernst, M. (2015). The CRH1 antagonist GSK561679 increases human fear but not anxiety as assessed by startle. Neuropsychopharmacology, 40, 1064–1071. doi: 10.1038/npp.2014.316 25430779 10.1038/npp.2014.316 PMC4367474

[psyp12545-bib-0036] Grillon, C. , Sinha, R. , Ameli, R. , & O'Malley, S. S. (2000). Effects of alcohol on baseline startle and prepulse inhibition in young men at risk for alcoholism and/or anxiety disorders. Journal of Studies on Alcohol and Drugs, 61, 46–54. doi: 10.15288/jsa.2000.61.46 10.15288/jsa.2000.61.46 10627096

[psyp12545-bib-0037] Hawk, L. W. , & Cook, E. W. (2000). Independence of valence modulation and prepulse inhibition of startle. Psychophysiology, 37, 5–12. doi: 10.1111/1469-8986.3710005 10705762

[psyp12545-bib-0038] Hogle, J. M. , & Curtin, J. J. (2006). Sex differences in negative affective response during nicotine withdrawal. Psychophysiology, 43, 344–356. doi: 10.1111/j.1469-2006.00406.x 16916430 10.1111/j.1469-8986.2006.00406.x

[psyp12545-bib-0039] Hogle, J. M. , Kaye, J. T. , & Curtin, J. J. (2010). Nicotine withdrawal increases threat‐induced anxiety but not fear: Neuroadaptation in human addiction. Biological Psychiatry, 68, 687–688. doi: 10.1016/j.biopsych.2010.06.003 20673878 10.1016/j.biopsych.2010.06.003 PMC2949532

[psyp12545-bib-0040] Jin, P. (1992). Toward a reconceptualization of the law of initial value. Psychological Bulletin, 111, 176–184. doi: 10.1037/0033-2909.111.1.176 1539087 10.1037/0033-2909.111.1.176

[psyp12545-bib-0041] Jovanovic, T. , Nylocks, K. M. , Gamwell, K. L. , Smith, A. , Davis, T. A. , Norrholm, S. D. , & Bradley, B. (2014). Development of fear acquisition and extinction in children: Effects of age and anxiety. Neurobiology of Learning and Memory, 113, 135–142. doi: 10.1016/j.nlm.2013.10.016 24183838 10.1016/j.nlm.2013.10.016 PMC4004724

[psyp12545-bib-0042] Kirk R. E. (Ed.) (1995). Multiple comparison tests In Experimental design: Procedures for the behavioral sciences (pp. 90–133). Pacific Grove, CA: Brooks/Cole.

[psyp12545-bib-0043] Koch, M. (1999). The neurobilogy of startle. Progress in Neurobiology, 59, 107–128. 10463792 10.1016/s0301-0082(98)00098-7

[psyp12545-bib-0044] Lang, P. J. (1995). The emotion probe: Studies of motivation and attention. American Psychologist, 50, 372–385. doi: 10.1037/0003-066X.50.5.372 7762889 10.1037//0003-066x.50.5.372

[psyp12545-bib-0045] Levenson, R. , Sher, K. , Grossman, L. , Newman, J. , & Newlin, D. (1980). Alcohol and stress response dampening: Pharmacological effects, expectancy, and tension reduction. Journal of Abnormal Psychology, 89, 528–538. doi: 10.1037/0021-843X.89.4.528 7400453 10.1037//0021-843x.89.4.528

[psyp12545-bib-0046] Levenston, G. K. , Patrick, C. J. , Bradley, M. M. , & Lang, P. J. (2000). The psychopath as observer: Emotion and attention in picture processing. Journal of Abnormal Psychology, 109, 373–385. doi: 10.1037/0021-843X.109.3.373 11016107

[psyp12545-bib-0047] Miller, M. W. , Curtin, J. J. , & Patrick, C. J. (1999). A startle probe methodology for investigating the effects of active avoidance on negative emotional reactivity. Biological Psychology, 50, 235–257. doi: 10.1016/S0301-0511(99)00011-3 10461807 10.1016/s0301-0511(99)00011-3

[psyp12545-bib-0048] Moberg, C. A. , & Curtin, J. J. (2009). Alcohol selectively reduces anxiety but not fear: Startle response during unpredictable vs. predictable threat. Journal of Abnormal Psychology, 118, 335–347. doi: 10.1037/a0015636 19413408 10.1037/a0015636 PMC2756160

[psyp12545-bib-0049] Myrtek, M. , & Foerster, F. (1986). The law of initial value: A rare exception. Biological Psychology, 22, 227–237. doi: 10.1016/0301-0511(86)90028-1 3756285 10.1016/0301-0511(86)90028-1

[psyp12545-bib-0050] Nelson, B. D. , McGowan, S. K. , Sarapas, C. , Robison‐Andrew, E. J. , Altman, S. E. , Campbell, M. L. , … Shankman, S. A. (2013). Biomarkers of threat and reward sensitivity demonstrate unique associations with risk for psychopathology. Journal of Abnormal Psychology, 122, 662–671. doi: 10.1037/a0033982 24016008 10.1037/a0033982 PMC3790143

[psyp12545-bib-0051] NIMH–Negative Valence Systems: Workshop Proceedings. (2011). Retrieved July 5, 2013, from http://www.nimh.nih.gov/research-priorities/rdoc/negative-valence-systems-workshop-proceedings.shtml

[psyp12545-bib-0052] Nosek, B. A. , Alter, G. , Banks, G. C. , Borsboom, D. , Bowman, S. J. , Breckler, S. J. , … Yarkoni, T. (2015). Promoting an open research culture. Science, 348, 1422–1425. doi: 10.1126/science.aab2374 26113702 10.1126/science.aab2374 PMC4550299

[psyp12545-bib-0053] Pelli, D. G. (1997). The VideoToolbox software for visual psychophysics: Transforming numbers into movies. Spatial Vision, 10, 437–442. 9176953

[psyp12545-bib-0054] R Development Core Team . (2013). R: A language and environment for statistical computing. Vienna, Austria: R Foundation for Statistical Computing Retrieved from http://www.R-project.org

[psyp12545-bib-0055] Rich, B. A. , Bhangoo, R. K. , Vinton, D. T. , Berghorst, L. H. , Dickstein, D. P. , Grillon, C. , … Leibenluft, E. (2005). Using affect‐modulated startle to study phenotypes of pediatric bipolar disorder. Bipolar Disorders, 7, 536–545. doi: 10.1111/j.1399-5618.2005.00265.x 16403179 10.1111/j.1399-5618.2005.00265.x

[psyp12545-bib-0056] Rodríguez‐Fornells, A. , Riba, J. , Gironell, A. , Kulisevsky, J. , & Barbanoj, M. J. (1999). Effects of alprazolam on the acoustic startle response in humans. Psychopharmacology, 143, 280–285. 10353431 10.1007/s002130050948

[psyp12545-bib-0057] Rohsenow, D. J. , & Marlatt, G. A. (1981). The balanced placebo design: Methodological considerations. Addictive Behaviors, 6, 107–122. doi: 10.1016/0306-4603(81)90003-4 7023202 10.1016/0306-4603(81)90003-4

[psyp12545-bib-0058] Schmitz, A. , Merikangas, K. , Swendsen, H. , Cui, L. , Heaton, L. , & Grillon, C. (2011). Measuring anxious responses to predictable and unpredictable threat in children and adolescents. Journal of Experimental Child Psychology, 110, 159–170. doi: 10.1016/j.jecp.2011.02.014 21440905 10.1016/j.jecp.2011.02.014 PMC3110515

[psyp12545-bib-0059] Sege, C. T. , Bradley, M. M. , & Lang, P. J. (2014). Startle modulation during emotional anticipation and perception. Psychophysiology, 51, 977–981. doi: 10.1111/psyp.12244 24980898 10.1111/psyp.12244 PMC4165748

[psyp12545-bib-0060] Sher, K. J. (1987). Stress response dampening In Blane H. T. & Leonard K. E. (Eds.), Psychological theories of drinking and alcoholism (pp. 227–271). New York, NY: Guilford Press.

[psyp12545-bib-0061] Sher, K. J. , Bartholow, B. D. , Peuser, K. , Erickson, D. J. , & Wood, M. D. (2007). Stress‐response‐dampening effects of alcohol: Attention as a mediator and moderator. Journal of Abnormal Psychology, 116, 362–377. doi: 10.1037/0021-843X.116.2.362 17516768 10.1037/0021-843X.116.2.362 PMC2679524

[psyp12545-bib-0062] Simmons, J. P. , Nelson, L. D. , & Simonsohn, U. (2011). False‐positive psychology undisclosed flexibility in data collection and analysis allows presenting anything as significant. Psychological Science, 22, 1359–1366. doi: 10.1177/0956797611417632 22006061 10.1177/0956797611417632

[psyp12545-bib-0063] Smith, J. C. , Bradley, M. M. , & Lang, P. J. (2005). State anxiety and affective physiology: Effects of sustained exposure to affective pictures. Biological Psychology, 69, 247–260. doi: 10.1016/j.biopsycho.2004.09.001 15925028 10.1016/j.biopsycho.2004.09.001

[psyp12545-bib-0064] Sripada, C. S. , Angstadt, M. , McNamara, P. , King, A. C. , & Phan, K. L. (2011). Effects of alcohol on brain responses to social signals of threat in humans. NeuroImage, 55, 371–380. doi: 10.1016/j.neuroimage.2010.11.062 21122818 10.1016/j.neuroimage.2010.11.062 PMC3031714

[psyp12545-bib-0065] Stern, R. M. , Ray, W. J. , & Quigley, K. S. (2001). Psychophysiological recording. Oxford, UK: Oxford University Press.

[psyp12545-bib-0066] Stritzke, W. G. K. , Patrick, C. J. , & Lang, A. R. (1995). Alcohol and human emotion: A multidimensional analysis incorporating startle‐probe methodology. Journal of Abnormal Psychology, 104, 114–122. doi: 10.1037/0021-843X.104.1.114 7897033 10.1037//0021-843x.104.1.114

[psyp12545-bib-0067] Swerdlow, N. R. , Geyer, M. A. , & Braff, D. L. (2001). Neural circuit regulation of prepulse inhibition of startle in the rat: Current knowledge and future challenges. Psychopharmacology, 156, 194–215. doi: 10.1007/s002130100799 11549223 10.1007/s002130100799

[psyp12545-bib-0068] Vaidyanathan, U. , Malone, S. M. , Miller, M. B. , McGue, M. , & Iacono, W. G. (2014). Heritability and molecular genetic basis of acoustic startle eye blink and affectively modulated startle response: A genome‐wide association study. Psychophysiology, 51, 1285–1299. doi: 10.1111/psyp.12348 25387708 10.1111/psyp.12348 PMC4231542

[psyp12545-bib-0069] Vaidyanathan, U. , Patrick, C. J. , & Cuthbert, B. N. (2009). Linking dimensional models of internalizing psychopathology to neurobiological systems: Affect‐modulated startle as an indicator of fear and distress disorders and affiliated traits. Psychological Bulletin, 135, 909–942. doi: 10.1037/a0017222 19883142 10.1037/a0017222 PMC2776729

[psyp12545-bib-0070] Vanman, E. J. , Mejia, V. Y. , Dawson, M. E. , Schell, A. M. , & Raine, A. (2003). Modification of the startle reflex in a community sample: Do one or two dimensions of psychopathy underlie emotional processing? Personality and Individual Differences, 35, 2007–2021. doi: 10.1016/S0191-8869(03)00052-7

[psyp12545-bib-0071] Walker, D. , & Davis, M. (2002). Quantifying fear potentiated startle using absolute versus proportional increase scoring methods: Implications for the neurocircuitry of fear and anxiety. Psychopharmacology, 164, 318–328. doi: 10.1007/s00213-002-1213-0 12424556 10.1007/s00213-002-1213-0

[psyp12545-bib-0072] Wilder, J. (1967). Stimulus and response: The law of initial value. Bristol, UK: Wright.

[psyp12545-bib-0073] Yancey, J. R. , Vaidyanathan, U. , & Patrick, C. J. (2014). Aversive startle potentiation and fear pathology: Mediating role of threat sensitivity and moderating impact of depression. International Journal of Psychophysiology. Advance online publication. doi: 10.1016/j.ijpsycho.2014.10.014 10.1016/j.ijpsycho.2014.10.014 PMC4422769 25448265

